# Assessing the perceived quality of brachial artery Flow Mediated Dilation studies for inclusion in meta-analyses and systematic reviews: Description of data employed in the development of a scoring ;tool based on currently accepted guidelines

**DOI:** 10.1016/j.dib.2016.05.011

**Published:** 2016-05-13

**Authors:** Arno Greyling, Anke C.C.M van Mil, Peter L. Zock, Daniel J. Green, Lorenzo Ghiadoni, Dick H. Thijssen, on behalf of the TIFN International Working Group on Flow Mediated Dilation

**Affiliations:** a Department of Physiology, Radboud University Medical Centre, Nijmegen, The Netherlands; b Unilever R&D Vlaardingen, Vlaardingen, The Netherlands; c TI Food and Nutrition, Wageningen, The Netherlands; d Maastricht University Medical Centre, Maastricht, The Netherlands; e School of Sports Science, Exercise and Health, The University of Western Australia, Crawley, Australia; f University of Pisa, Pisa, Italy; g Research institute for Sport and Exercise Sciences, Liverpool John Moores University, Liverpool, United Kingdom

**Keywords:** Cardiovascular disease, Atherosclerosis, Endothelial function, Reproducibility, Methodology

## Abstract

Brachial artery Flow Mediated Dilation (FMD) is widely used as a non-invasive measure of endothelial function. Adherence to expert consensus guidelines on FMD measurement has been found to be of vital importance to obtain reproducible data. This article lists the literature data which was considered in the development of a tool to aid in the objective judgement of the extent to which published studies adhered to expert guidelines for FMD measurement. Application of this tool in a systematic review of FMD studies (http://dx.doi.org/10.1016/j.atherosclerosis.2016.03.011) (Greyling et al., 2016 [1]) indicated that adherence to expert consensus guidelines is strongly correlated to the reproducibility of FMD data.

**Specifications Table**

**Table:** 

Subject area	*Medicine*
More specific subject area	*Vascular Physiology*
Type of data	*Table*
How data was acquired	*Systematic literature survey and expert consensus*
Data format	*Processed*
Experimental factors	*Methodological parameters related to valid measurement FMD*
Experimental features	*Assessment tool based on 33 studies pertaining to the most appropriate methods to assess FMD in humans identified from literature and expert guidelines for FMD measurement*
Data source location	*Nijmegen, The Netherlands*
Data accessibility	*Data is within this article*

## **Value of the data**

• The literature data provided here establishes an evidence base and a physiological background rationale for the individual components included in the Adherence Score, aiding in the improvement of the practical guidance and technical approaches to FMD measurement and analysis.
• This “Adherence Score” which ranges between 0 (i.e. no adherence) and 10 (i.e. full adherence) can conceivably be employed to evaluate the perceived quality of studies reporting FMD data, with a higher outcome of this measure being strongly related to better reproducibility of the FMD data [1].
• This tool may prove useful additional information when pooling, contrasting and comparing different studies, e.g. for the purpose of meta-analyses or systematic reviews.

## 1 Data

A tool to enable objective assessment of the level adherence to the FMD guidelines was developed. Table 1 presents the 19 different factors that make up the *“Adherence Score”* tool along with citations to the literature data which justify the inclusion of each factor in question.

## 2 Experimental design, materials and methods

Based on previous expert-consensus guidelines [35], we devised a scoring system reliant on the reporting of 19 different methodological factors related to FMD measurement. These factors were identified after critical review and appraisal of published physiological studies pertaining to the most appropriate methods to assess FMD in humans. Values were assigned to each component proportional to its perceived importance for valid assessment of the FMD. This was done through expert consensus discussion within the Working Group (AG, LG and DHJT). The *“Adherence Score”* that any given study can be assigned ranges from 0 to 10 points depending on how many of the 19 different factors that are reported or referred to in the text of the paper in question.

## Figures and Tables

**Table 1:** Scoring tool based on currently accepted guidelines for the assessment of the perceived quality of FMD studies [2], [3], [4], [5], [6], [7], [8], [9], [10], [11], [12], [13], [14], [15], [16], [17], [18], [19], [20], [21], [22], [23], [24], [25], [26], [27], [28], [29], [30], [31], [32], [33], [34].
